# Application of Convolutional Recurrent Neural Network for Individual Recognition Based on Resting State fMRI Data

**DOI:** 10.3389/fnins.2019.00434

**Published:** 2019-05-01

**Authors:** Lebo Wang, Kaiming Li, Xu Chen, Xiaoping P. Hu

**Affiliations:** ^1^Department of Electrical and Computer Engineering, University of California, Riverside, Riverside, CA, United States; ^2^Department of Bioengineering, University of California, Riverside, Riverside, CA, United States; ^3^Center for Advanced Neuroimaging, University of California, Riverside, Riverside, CA, United States

**Keywords:** functional magnetic resonance imaging, individual identification, recurrent neural network, convolutional neural network, visualization

## Abstract

In most task and resting state fMRI studies, a group consensus is often sought, where individual variability is considered a nuisance. None the less, biological variability is an important factor that cannot be ignored and is gaining more attention in the field. One recent development is the individual identification based on static functional connectome. While the original work was based on the static connectome, subsequent efforts using recurrent neural networks (RNN) demonstrated that the inclusion of temporal features greatly improved identification accuracy. Given that convolutional RNN (ConvRNN) seamlessly integrates spatial and temporal features, the present work applied ConvRNN for individual identification with resting state fMRI data. Our result demonstrates ConvRNN achieving a higher identification accuracy than conventional RNN, likely due to better extraction of local features between neighboring ROIs. Furthermore, given that each convolutional output assembles in-place features, they provide a natural way for us to visualize the informative spatial pattern and temporal information, opening up a promising new avenue for analyzing fMRI data.

## Introduction

Mainstream fMRI studies have been focusing on deriving population consensuses using group analysis. A group analysis in neuroimaging, albeit important, commonly neglects individual-to-individual variations. The importance of individual variability in neurobiological research has drawn increasing attention (Mohr and Nagel, 2010). Using task-fMRI, significant individual differences in brain activation were identified, reflecting alterations in cognitive function and behavior (Barch et al., 2013). Individual variability in functional connectivity (FC) has been successfully used to identify subjects from a large group. More specifically, static connectivity patterns throughout the brain were shown to be subject specific and distinctive across scan sessions and conditions, providing powerful features for individual identification (Finn et al., 2015). Therefore, exploring the individual uniqueness of the brain connectivity points to a new avenue to study the brain.

Although the static FC achieved decent accuracy, it required a sufficiently long data set (600 frames, 7.2 min) and considered only the spatial pattern through the temporal correlation without taking temporal features into full account. The performance degraded with short clips of fMRI data, probably due to temporal variability (or dynamics) in the resting-state fMRI data, which leads to high variability in the FC derived from a short window. On the other hand, the dynamic information of resting state activity, if taken into account, could provide additional features for individual identification, improving the accuracy with the short time series.

In the application of time sequence modeling, recurrent neural networks (RNNs) have shown outstanding promise in a broad range of applications, including video classification, machine translation, and biomedical image segmentation (Sharma et al., 2015; Chen et al., 2016; Vaswani et al., 2017; Gao et al., 2018). For fMRI data analysis, RNN was able to model the dynamics of brain activity in response to sensory stimuli, providing accurate estimates of hemodynamic response with temporal dynamics (Güçlü and van Gerven, 2017). RNNs have also been implemented to incorporate temporal information along with spatial features from resting-state fMRI data instead of merely spatial pattern in the connectome (Dvornek et al., 2017; Chen and Hu, 2018). Furthermore, a convolution-based RNN was introduced to make full use of features in both spatial and temporal domains, consistently outperforming fully connected RNNs (Shi et al., 2015). Therefore, combining the local features between adjacent ROIs by the convolutional structure and sequence modeling capability of RNN may lead to a better approach to extract spatiotemporal features for individual identification on resting-state fMRI data.

In the meantime, it is also valuable to visualize the underlying features in the trained convolutional models. Although deep learning is becoming a panacea in almost every domain, it has been criticized due to its poor interpretability as being a black-box. While many attempts have been made to provide an interpretation and an intuitive understanding of trained networks, our understanding of how these networks work and what is important behind their performance have not kept up with the pace of the development of neural networks. While dedicated deep learning models have achieved amazing performance by end-to-end learning through huge volumes of data, better comprehension of the success of these models can uncover fundamental principles of deep neural networks and reveal important features within the data.

In this work, we adopted convolutional RNN or ConvRNN for individual identification using resting-state fMRI data. The convolutional recurrent model was able to achieve individual identification with shared convolutional weights capturing local coactivation features. In-place visualization of the informative area by ConvRNN also opened up a new avenue for understanding fMRI data based on individual differences.

## Materials and Methods

### Dataset and Preprocessing

**FIGURE 1:**
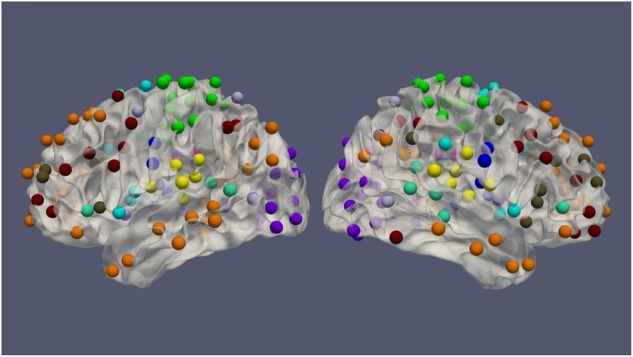
The spatial distribution of 236 ROIs over the cerebral cortex. Voxels within the 10 mm diameter sphere were averaged to get the value for each ROI.

The resting-state fMRI data for 100 subjects from the Human Connectome Project (HCP) (54 females, age: 22–36, and TR = 0.72 s) was used in this work. Each subject had four resting-state fMRI sessions, 1200 volumes for each session, leading to 4800 volumes per subject in total (Van Essen et al., 2013). The fMRI data was preprocessed by the HCP minimal preprocessing pipeline (Glasser et al., 2013) and denoised by ICA-FIX (Salimi-Khorshidi et al., 2014), for the removal of spatial artifact/distortion and motion-related fluctuations. Surface-based registration was performed with the MSM-ALL template (Robinson et al., 2014). To decrease the computation complexity, two hundred and thirty-six regions of interest (ROIs) over the cerebral cortex, as shown in Figure 1 based on meta-analysis (Power et al., 2011), were used for subsequent analysis. BOLD signals within each ROI (10 mm diameter sphere) were averaged spatially. We ordered our ROIs-based data in a 1D array and preserved the order of ROIs according to the Power Atlas, where ROIs having similar functional connectivities are close to each other. They were also demeaned and scaled to unit variance over the temporal axis. For each fMRI session, fMRI data with 1200 volumes was divided into twelve 100-frame clips as inputs of ConvRNN. Data from Day 1 was used as the training dataset. The two sessions from Day 2 were used as validation and testing datasets, respectively. The best model was decided based on the validation dataset and the final performance was assessed on the testing dataset.

### Convolutional Recurrent Neural Network

**FIGURE 2:**
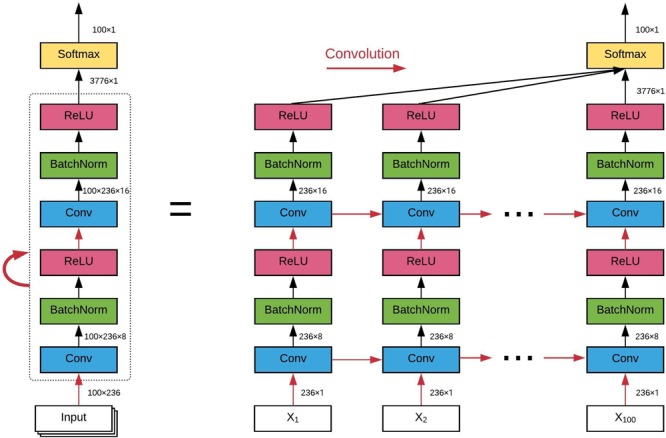
The architecture of our convolutional recurrent neural network and its unrolling version over time. All red arrows represent the convolutional operations between each input-to-state and state-to-state transitions. Batch normalization and ReLU as the non-linear activation are utilized after each convolutional layer. Final classification is based on all hidden states on average. The dimension of the data flow through the diagram is also labeled.

The architecture of the ConvRNN is given in Figure 2, along with its unrolling version. In contrast to conventional RNN, convolution was applied in both the input-to-state and state-to-state transitions, in place of the Hadamard product. There were two stacked convolutional layers, with the first convolutional layer containing 8 filters and the second convolutional layer having 16 filters. The kernel size of all convolutional filters was 2. Padding was used in all convolutional layers such that the outputs from each layer had the same spatial dimension as the original input, which is very important for the subsequent visualization of the in-place features. Batch normalization layers were used before the non-linear activation layers of Rectified linear unit (ReLU) to reshape the distribution of convolutional layer outputs in order for better convergence and easy training (Ioffe and Szegedy, 2015). The final Softmax layer with 100-category outputs was used for classification based on averaged outputs from all hidden states. No temporal or spatial pooling layer was employed to keep the spatial and temporal resolutions of the original fMRI data. All kernel weights were initialized by the Xavier uniform initializer (Glorot and Bengio, 2010), and recurrent weights were initialized as random orthogonal matrices (Saxe et al., 2013).

### Training of the Neural Network

Our implementation of ConvRNN was carried out in Keras (Chollet, 2015) with the Tensorflow backend (Abadi et al., 2016). Considering the limited number of frames for each subject, we chose 100 frames of fMRI data as inputs during training and validation, which is the tradeoff between the number of fMRI clips and the number of frames for each clip. Shuffled minibatches of training data as inputs were fed into the ConvRNN with the batch size of 128. Adam optimizer (Kingma and Ba, 2014) was applied for training with the initial learning rate set to 0.001, and reduced if the validation accuracy stopped increasing. Dropout layer with 50% was utilized before the final classification to avoid overfitting only during the training (Srivastava et al., 2014). After each training epoch, the model was evaluated on the validation dataset and saved only if better validation accuracy was achieved. Finally, the performance of the best model was measured on the testing dataset, which was never involved during training or validation.

It is well known that RNN is difficult to train properly, even though it is a powerful model for time series modeling. The main reasons are vanishing and exploding gradient issues of Backpropagation Through Time (BPTT) on the unrolling version of RNN (Bengio et al., 1994). Therefore, advanced architectures with gating mechanism to overcome the vanishing and exploding gradient problem, such as the Long short-term memory (LSTM) (Hochreiter and Schmidhuber, 1997) and the Gated recurrent unit (GRU) (Cho et al., 2014), have gained a lot of popularity in practice to model long-term dependencies. In this work, LSTM with convolutional structure was applied. For training techniques, we used L2 regularization for recurrent weights, along with the gradient clipping strategy as a simple and computationally efficient method, effectively addressing the issue of exploding gradients (Pascanu et al., 2013). In the present work, the clipping norm of the gradient was set to 1. Different L2 values (0.1, 0.01, 0.001, and 0.0001) on recurrent kernel weights were tested to achieve the best validation accuracy.

### Visualization of the Individual Identification

Our ConvRNN first performed feature extraction through two convolutional recurrent layers and then fed the features into the Softmax layer for the 100-catergory classification. Original data was projected to a high-dimensional feature space, which was easily separated by the classification layer. In the feature space, fMRI data from the same subject are expected to be close to each other and cluster tightly. In consideration of the single classification layer, the identification accuracy of our ConvRNN relies heavily on the performance of feature extraction by convolutional recurrent layers. In order to ascertain and visualize the performance of convolutional recurrent layers in low dimensional space, t-Distributed Stochastic Neighbor Embedding (t-SNE) (Maaten and Hinton, 2008) was applied to map datapoints in high-dimensional feature space onto a two-dimensional representation.

To visualize and understand informative areas related to individual identification, intermediate outputs from convolutional layers were examined. Output patterns were obtained from convolutional layers after non-linear activation and mapped onto the cortical surface (20 mm radius sphere). As all regions are considered equal in our convolutional model during the training, but they are of different importance to the final classification. We also used the occlusion method to visualize informative areas (Zeiler and Fergus, 2014). More specifically, in order to ascertain the contribution of ROIs with regard to individual identification, input of each ROI was zeroed out, and the subsequent performance decrease with the same model configuration was considered as the contribution of this ROI to the final classification.

## Results

**FIGURE 3:**
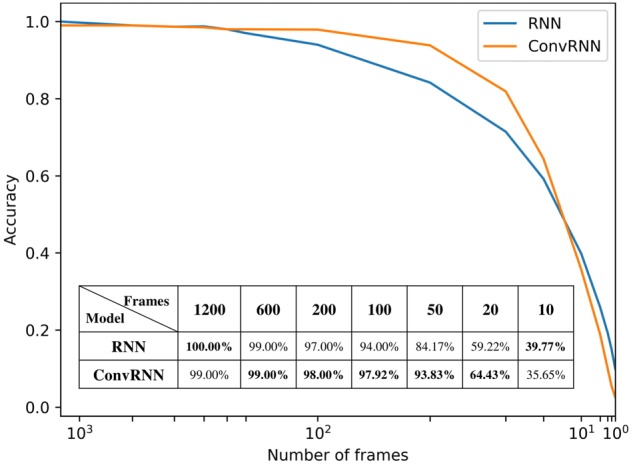
The relationship between identification accuracy and the window size. We evaluated pre-trained models on testing dataset. Our ConvRNN outperformed RNN except with 1200 frames or with less than 10 frames.

We carried out the supervised classification task to identify each subject from a group of 100 subjects. First, the identification accuracy of different models was assessed on the testing dataset with 100 frames of fMRI data as inputs. As seen in Table 1, our ConvRNN model was able to achieve 98.50% accuracy on the testing dataset, where the best performance was obtained with the L2 value of 0.001 during training. The test accuracy for the traditional RNN with average temporal pooling was 94.43% (Chen and Hu, 2018). In order to exclude the influence of temporal averaging, we trained another RNN without the temporal averaging and achieved an identification accuracy of 95.33%. Furthermore, we evaluated the performance of these models using different window sizes on the testing dataset. With the pre-trained models, we adopted different number of frames (from 1200 frames to single frame) as inputs from the testing dataset and evaluated the identification performance. Testing results with different number of frames are plotted in Figure 3. As shown in the figure, ConvRNN outperformed conventional RNN (no temporal averaging) in all cases except with 1200 frames or with less than 10 frames. In contrast, FC could achieve over 90% accuracy with 600 frames of fMRI data. But the individual identification accuracy drops to 70% on average when only a short period of fMRI data (100 frames) is used (Finn et al., 2015).

**Table 1:** The accuracy of different models on the testing dataset and their number of model weights.

Architecture	# Parameters	Test accuracy
	(feature extraction)	
RNN (Chen and Hu, 2018)	405K (380K)	94.43%
RNN w/o temporal pooling	405K (380K)	95.33%
ConvRNN	382K (3.8K)	98.50%

**FIGURE 4:**
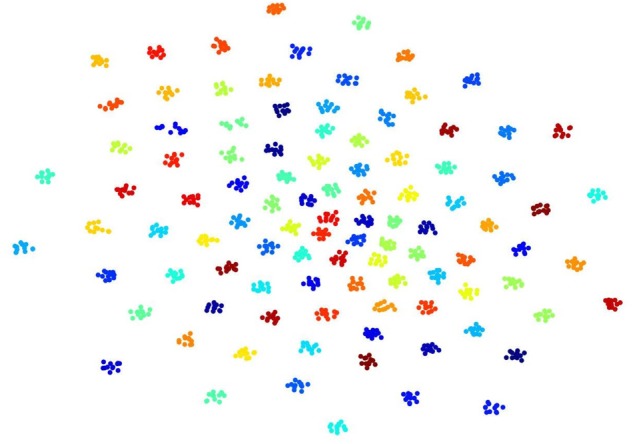
t-Distributed Stochastic Neighbor Embedding (t-SNE) visualization of 2nd convolutional recurrent layer outputs based on 100-subject testing dataset. Twelve hundred 100-frame testing data from 100 subjects were fed into ConvRNN with outputs being obtained before the classification layers and projected to 2D space by t-SNE. Projections for different subjects are in different colors.

To visualize convolutional outputs on low-dimensional space, we applied t-SNE on intermediate outputs of our ConvRNN before the classification layer. There were 16 convolutional filters in the second convolutional recurrent layer of our ConvRNN. With proper padding, the output of the layer was made to have the same spatial dimension as the input. The feature space with 3776 dimensions was then mapped to a 2D space in Figure 4. It is clear that 100 subjects (12 clips with 100 frames for each subject) in the testing dataset appear as non-overlapping cliques in different colors with each clique representing one subject. This figure clearly indicates that spatiotemporal features, capable of individual identification, were successfully obtained by convolutional recurrent layers.

**FIGURE 5:**
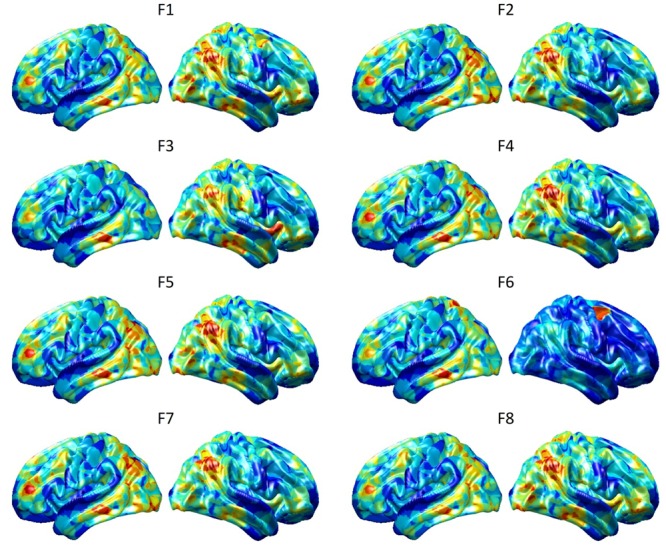
Average output patterns of the first convolutional layer with 8 convolutional kernels. Twelve hundred 100-frame testing data from 100 subjects were fed into the convolutional recurrent model with output patterns generated and averaged from the first non-linear activation layer. Red areas represent large activation values.

**FIGURE 6:**
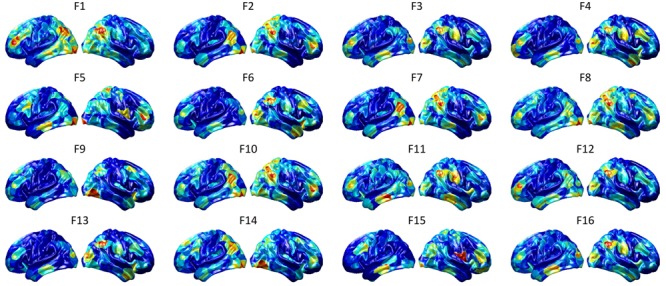
Average output patterns of the second convolutional layer with 16 convolutional kernels. Twelve hundred 100-frame testing data from 100 subjects were fed into the convolutional recurrent model with output patterns generated and averaged from the second non-linear activation layer. Red areas represent large activation values.

**FIGURE 7:**
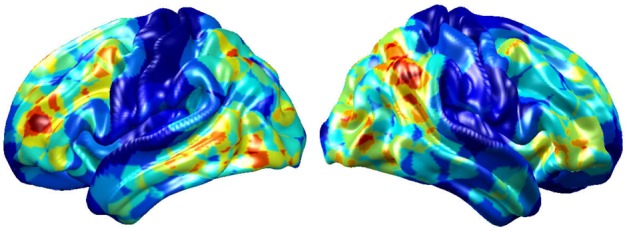
The performance degradation with occlusion. Each ROI was zeroed out separately and evaluated with the pre-trained model of ConvRNN. The performance degradation reflects the contribution of each ROI. Red region reflects large performance degradation if corresponding ROIs were occluded.

To visualize intermediate outputs of ConvRNN, average patterns from first and second convolutional layers of ConvRNN are shown in Figure 5, 6, respectively. Most patterns from the first convolutional layer were quite similar (except Filter 6) with large distinctive areas, which could be considered as the ubiquitous low-level features from fMRI data. While high-level patterns from the second convolutional layer had diverse informative regions, which were sparse and localized inside the area of those low-level features generated by the first convolutional layer. Meanwhile, when ROIs were individually occluded, performance degradation was observed when some ROIs were occluded, while the occlusion of some ROIs led to negligible degradation of the performance. In Figure 7, the absolute value of the performance degradation, normalized to reflect the contribution of each ROI is shown. It is evident that the informative area generated by alternative occlusion was similar as the patterns from first convolutional layer of ConvRNN.

## Discussion

While most resting-state fMRI studies have relied on group averages, this study employed individual differences for individual identification. Unlike the first study of individual identification employing static FC (Finn et al., 2015), we incorporated both temporal and spatial features from the fMRI data. As an improvement of our previous work employing the recurrent architecture (Chen and Hu, 2018), we applied a convolutional recurrent neural network which led to a significant improvement in performance and a straightforward means to visualize in-place features. Figure 3 shows that ConvRNN is better than conventional RNN for the majority of the time windows. The performance of ConvRNN was slightly worse than conventional RNN with 1200 frames, probably due to the small number of testing data when the performance was evaluated on fMRI clips with 1200 frames. On the other hand, since our ConvRNN was trained with the fixed number of frames (i.e., 100), it is not be optimized for short clips of data with less than 10 frames, and its performance with frames less than 10 is therefore worse than that of conventional RNN.

Our ConvRNN has the same number of parameters compared with the conventional RNN, indicating that both models have comparable model complexity. Apart from the different types of recurrent unit, our earlier work (Chen and Hu, 2018) employed a temporal pooling layer to reduce the temporal resolution. For a fair comparison with this work, another conventional RNN was applied without the temporal averaging layer. The accuracy of the conventional RNN without the temporal averaging layer was 95.33%, which was 0.9% higher than that with the temporal averaging. This improvement due to increased temporal resolution is significantly smaller than the improvement achieved with the adoption of the convolutional structure, indicating that the latter is the main contributor to the performance enhancement. Both the spatial and temporal features were fully utilized by convolutional kernels in the ConvRNN with unprecedented identification accuracy. Also, feature extraction layers of ConvRNN showed strong discriminating power for 100 subjects, where pre-trained layers could be applied for transfer learning on new subjects or semi-supervised learning on partially labeled data. On the other hand, only one hundredths of the parameters in ConvRNN were from the convolutional recurrent layers. Convolution with shared weights in spatial and temporal spans makes it more robust against overfitting during training. Given the reduced number of trainable weights, increasing the depth and width of the model is possible without significantly increasing the model complexity, possibly capturing more sophisticated features in both spatial and temporal domains.

Furthermore, convolutional kernels with shared weights sweep across ROIs and frames. Different from the indecomposable matrix multiplication in conventional RNN, ConvRNN generates in-place features with exactly spatial correspondence as the original data. Furthermore, ConvRNN accumulates temporal information related to evolving features in the hidden state. Therefore, it is possible to examine the hidden states to have an in-place visualization and understanding of hidden features from ConvRNN. It is also clear that informative regions from two convolutional layers are totally different, in agreement with the conclusion drawn from convolutional neural networks for image classification (Yosinski et al., 2015). Beside the direct visualization of the hidden state, the occlusion of ROIs served as an indirect method for visualizing significant regions under resting state for identification. Two visualization approaches came to the same conclusion of informative ROIs in term of individual identification using the resting-state fMRI data. In terms of resting state networks (RSNs) in the literature (Holmes et al., 2011; Lee et al., 2012), the informative area identified by our ConvRNN mainly contained frontoparietal network (FPN), default mode network (DMN), as well as visual network (VN). Our result is consistent with a previous study, which concluded that the most distinguishing network was FPN, with significant improvement achieved through the combination of multiple RSNs (Finn et al., 2015). In contrast, occlusion of language network (LN) and somatosensory motor network (SMN) did not cause much reduction in performance. One possible explanation is that there was little explicit or individual-specific language or motor activity during the acquisition of the resting-state fMRI datasets used here.

Although DMN and FPN stood out as the most important networks for individual identification, other networks also contributed to individual identification (Finn et al., 2017). It is likely that contributions from most networks may be needed with more subjects to be identified. In addition, a hierarchical approach, incorporating all networks, might be the most appropriate and robust approach. Furthermore, a recent study demonstrated that task induced changes in FC provided better prediction of individuals, whereas resting-state fMRI data failed to capture the full range of individual differences (Greene et al., 2018). Such changes in FC could also be incorporated into our model to further improve the identification accuracy.

Several limitations should be noted for further work. First, the present study adopted only 236 ROIs within 10 mm diameter spheres on average, which was enough for an accurate identification for a group of 100 subjects. Inadequate power of identification could be present on new subjects beyond existing subjects. Possible reasons are the limited feature extraction of our model and the high variability of the fMRI data. It is likely that smaller and more ROIs are needed for the identification of more subjects. But more advanced models with good generalization on fMRI data should be explored. Second, current visualization of individual identification depicted in Figure 7 was based on group average of ROIs’ performance. While this highlights areas that are most important for individual identification, it does not explicitly depict individual features. Such features will be the focus of our future studies. Third, visualization of the spatial pattern is easy to understand, but the remarkable performance achieved by RNN suggests that a substantial amount of information is coming from temporal features. Visualizing and understanding temporal features are still necessary to gain a deeper insight into the brain dynamics. Furthermore, other popular architectures (e.g., Siamese network) and pre-trained models should be applied and compared with our approach in terms of classification performance and training efficiency in future work.

## Conclusion

In this paper, we described the application of the convolutional recurrent neural network for individual identification based on resting-state fMRI data. To explore the dynamics in the resting-state fMRI data, the convolutional architecture with recurrent structure was implemented to extract and incorporate features in both spatial and temporal domains. Compared to conventional RNN model, our ConvRNN model exhibited better identification performance, with local features between neighboring ROIs being modeled by convolutional kernels. Moreover, visualization based on the ConvRNN model provides a direct understanding of the success of identification; this could lead to a promising alternative for analyzing fMRI data.

## Author Contributions

LW designed and performed experiments and wrote the manuscript. KL analyzed data and reviewed the manuscript. XC collected the data. XH designed research and reviewed the manuscript.

## Conflict of Interest Statement

The authors declare that the research was conducted in the absence of any commercial or financial relationships that could be construed as a potential conflict of interest.
